# Non-linear Associations Between Visceral Adipose Tissue Distribution and Anthropometry-Based Estimates of Visceral Adiposity

**DOI:** 10.3389/fnut.2022.825630

**Published:** 2022-03-24

**Authors:** Aldo Scafoglieri, Jona Van den Broeck, Erik Cattrysse, Ivan Bautmans, Steven B. Heymsfield

**Affiliations:** ^1^Experimental Anatomy Research Department, Vrije Universiteit Brussel, Brussels, Belgium; ^2^Frailty in Aging Research Department, Vrije Universiteit Brussel, Brussels, Belgium; ^3^Pennington Biomedical Research Center, Baton Rouge, LA, United States

**Keywords:** visceral adipose tissue, subcutaneous adipose tissue, skeletal muscle, waist-to-hip ratio, waist circumference, nonlinearity

## Abstract

**Background:**

Recent evidence suggests that excess visceral adipose tissue (VAT) is associated with future loss of subcutaneous adipose tissue (SAT) and skeletal muscle (SM) with aging. In clinical settings (abdominal) circumferences are commonly used to estimate body composition (BC). We aimed to study the linearity of VAT distribution ratios (i.e., VAT/SAT ratio and VAT/SM ratio), waist-to-hip ratio (WHR) and waist circumference (WC) with age and the relationship of VAT distribution ratios with anthropometry (i.e., WHR and WC).

**Materials and Methods:**

BC was determined using whole body magnetic resonance imaging in a large multi-ethnic group of 419 adults (42% white, 30% black, 15% Hispanic, 13% Asian, 1% other) with a BMI ranging from 15.9 to 40.8kg/m^2^. Linear and non-linear regression analysis was used to examine the linearity of VAT distribution ratios and anthropometry from 18 to 88 years. The relation between VAT distribution ratios and anthropometry was assessed separately.

**Results:**

In both sexes non-linear relationships were found between BC estimates and age, and between BC measures mutually. The ratios of VAT/SAT and VAT/SM showed quadratic relationships with age. VAT distribution ratios showed exponential or quadratic relationships with anthropometry with coefficients of determination ranging between 18 and 55%.

**Conclusion:**

In both sexes, VAT distribution ratios showed curvilinear relationships with age and with anthropometry. Given the sex differences in VAT distribution ratios, WHR and WC represent different BC proportions in men and women. These results emphasize the challenge when interpreting changes in BC based upon linear extrapolations in clinical practice.

## Introduction

The redistribution of tissues with aging, including the accumulation of ectopic fat in and around organs, has been related to the development of a variety of clinical disorders (1, 2). Aging induces changes in body composition (BC), such as an increase in visceral adipose tissue (VAT) and reduced muscle mass (3). Recently, it has been suggested that the accumulation of VAT precedes future loss of subcutaneous adipose tissue (SAT) and skeletal muscle (SM) (4–8). Understanding the intricate cross-talk between VAT and other tissue compartments may help clinicians in the screening for morbidity (9–12). Although still under investigation, the role of inflammation with aging has been brought forward as an important underlying mechanism in the development of cardiovascular and muscular dysfunction (13, 14). As a result, visceral adiposity and sarcopenic visceral obesity have been linked to adverse health outcomes (15–18) and negative survival (19–22).

Despite the accessibility to gold standard BC techniques in the context of potential life threatening health conditions such as diabetes or cancer, the need for simple, clinically applicable tools that can monitor changes in visceral and muscle tissue distribution over time remains topical. In clinical settings, the use of anthropometry-based markers of central obesity are widely accepted (23), but are not routinely obtained to determine disease risk (24–26). Although their ability to discriminate changes in VAT has previously been questioned (27, 28), there is a renewed interest in the use of waist circumference (WC) in the context of sarcopenic obesity (29, 30). Visceral adiposity and muscle mass change in opposite ways with aging (31), whereas anthropometry-based markers of central obesity gradually increase during lifespan (32–35). In order to advance our understanding of BC changes with aging (e.g., in the context of the operative definition of sarcopenic obesity) in clinical practice, the relation between VAT distribution ratios (i.e., VAT/SAT ratio, VAT/SM ratio), and anthropometry may be further explored (10, 36).

Previous studies investigating the relationship between VAT distribution, waist-to-hip (WHR) or WC have primarily considered a linear analytic approach (34). Investigating whether non-linear relations exist between different levels of organization in BC might help the interpretation of clinical results during screening for disease risk. Therefore we aimed to assess the linearity of VAT distribution ratios, WHR and WC to age in a multi-ethnic sample of healthy adults. Further, we assessed the linearity of VAT distribution ratios with WHR and WC in both sexes.

## Materials and Methods

### Participants

Four hundred and nineteen healthy women (*n* = 224) and men (*n* = 195) varying in ethnicity and age were recruited amongst hospital employees of St. Luke’s/Roosevelt Hospital (NY), students from Queen’s University (ON) and the general public of Kingston and New York. The study was ethically approved by the respective institutional review boards. All participants signed a written informed consent form prior to enrollment. None of the participants reported medication intake known to affect body composition. The study procedures were in accordance with the World Medical Association’s Declaration of Helsinki.

### Magnetic Resonance Imaging Segmentation

The participants lay in prone position with their arms placed straight overhead in a 1.5 Tesla scanner (GE, Milwaukee, WI). MRI images were obtained with a T1-weighted, spin-echo sequence with a 210-ms repetition time and a 17-ms echo time. The details of the MRI protocol are described elsewhere (37, 38). A total of approximately 40 images with a slice thickness of 1 cm were acquired from each participant. The MRI data were analyzed using a semi-automatic software program for segmentation (Tomovision, Montreal, PQ). This software program allowed for the discrimination between adipose and muscle tissues based on their gray-level histogram output and a watershed algorithm for the selection of VAT. After segmentation a highly trained analyst specialized in tissular anatomy visually inspected and edited all images.

### Body Composition Calculations

The volume of adipose and muscle tissue was calculated by multiplying the area in each image by the slice thickness. The volume of adipose and muscle tissue for the space between two consecutive slices was calculated with the use of a mathematical algorithm given elsewhere (38). The volume of tissues was converted to mass by multiplying the former by its assumed density. Densities of 0.92 g/cm^3^ and 1.04 g/cm^3^ were assumed for adipose tissue and muscle, respectively (39).

### Visceral Adipose Tissue Distribution Ratios

Visceral adipose tissue, total body SAT and total body SM were used to compute VAT distribution ratios. The ratio of VAT to SAT and the ratio of VAT to SM were used as metrics of VAT distribution.

### Anthropometric Variables

Body mass was measured in minimal clothing on a digital scale to the closest 0.1 kg. Stature was recorded to the nearest 5 mm using a wall-mounted stadiometer according to standard procedures. Waist and hip circumference were taken to the nearest 1 mm with an anthropometric steel measuring tape. Waist circumference was measured midway between the iliac crest and lower rib border and hip circumference at the greatest protuberance of the gluteal muscles.

### Statistical Analysis

Data were analyzed using MedCalc^®^ Statistical Software version 20.015 (MedCalc Software Ltd, Ostend, Belgium^1^). Data are expressed as group median [interquartile range] or mean ± standard deviation. Differences between women and men were tested for significance by unpaired *t*-tests or Mann-Whitney U tests, and for BC parameters by way of analysis of covariance (ANCOVA). Within each sex the linearity of the relation between VAT ratios, anthropometric variables and age was evaluated using linear and non-linear regression modeling based on least squares. An automatic weighted regression procedure was selected to correct for heteroscedasticity. The 0.05 level of significance was used for all data analyses.

## Results

The characteristics of the participants can be found in Table 1. The ethnicity of the participants was equally divided between both sexes: Asian (13%), black (30%), Hispanic (15%), white (42%) and other (1%). Sixty-three percent of women were in the premenopausal period. After correction for age, height and weight, all BC variables were significantly different between sexes, except for BMI.

**TABLE 1:** Characteristics of the participants.

	Women (*n* = 224)	Men (*n* = 195)
Age (years)	43.0 [32.0–56.0]	37.0 [28.0–47.0]^†^
Weight (kg)	64.9 [54.7–77.5]	80.0 ± 12.7^†^
Height (cm)	161.8 ± 7.2	176.9 ± 7.0^†^
BMI (kg/m^2^)	24.5 [21.6–29.0]	25.1 [22.7–28.2]
WHR	0.78 [0.74–0.83]	0.87 [0.83–0.92]^†^
WC (cm)	78.0 [69.4–88.5]	87.6 ± 10.7^†^
AT (kg)	21.9 [16.4–32.6]	17.1 [12.4–23.2]^†^
VAT (g)	985.0 [535.0–2105.5]	1679.4 [770.0–3372.5]^†^
SAT (kg)	20.9 [15.6–31.2]	15.0 [10.8–19.9]^†^
SM (kg)	19.7 [17.5–21.9]	31.7 ± 5.5^†^
VAT/SAT	4.8 [3.2–7.8]	11.1 [6.7–18.3]^†^
VAT/SM	5.2 [2.9–10.1]	5.8 [2.4–10.2]*

Data are expressed as median [interquartile rage] or mean ± standard deviation, WC = waist circumference, WHR = waist-to-hip ratio, AT = total body adipose tissue, VAT = visceral adipose tissue, SAT = total body subcutaneous adipose tissue, SM = total body skeletal muscle mass, *p < 0.01

*^†^p < 0.001, body composition variables were corrected for age, weight and height (ANCOVA).*

### Relationships of Body Composition With Age

**FIGURE 1:**
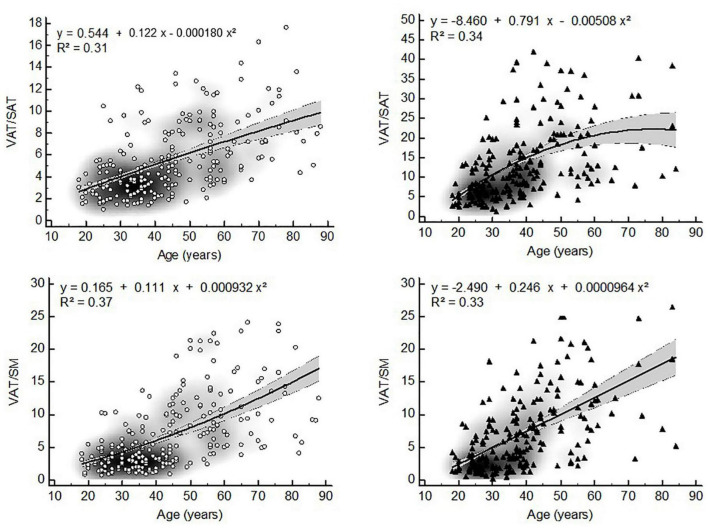
Non-linear relationships between VAT distribution ratios and age (▲ = women, ▲ = men, VAT = visceral adipose tissue, WHR = waist-to-hip ratio, WC = waist circumference, gray zone represents 95% confidence interval).

**FIGURE 2:**
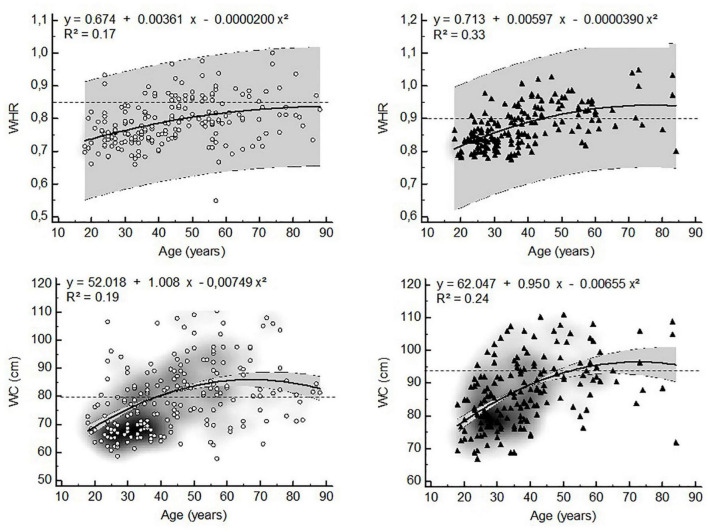
Non-linear relationships between anthropometric estimates of visceral adiposity and age (▲ = women, ▲ = men, WHR = waist-to-hip ratio, WC = waist circumference, dashed lines represent WHO (25) cut points for increased cardiometabolic risk in whites, gray zone represents 95% confidence interval).

Visceral adipose tissue distribution ratios, WHR and WC were non-linearly related to age in both sexes. The models with the best fit showed that VAT distribution ratios were quadratically related to age in both sexes (Figure 1). Similarly WHR and WC showed quadratic relationships with age (Figure 2).

### Relationships of Visceral Adipose Tissue Distribution Ratios With Waist Circumference and Waist-to-Hip Ratio

**FIGURE 3:**
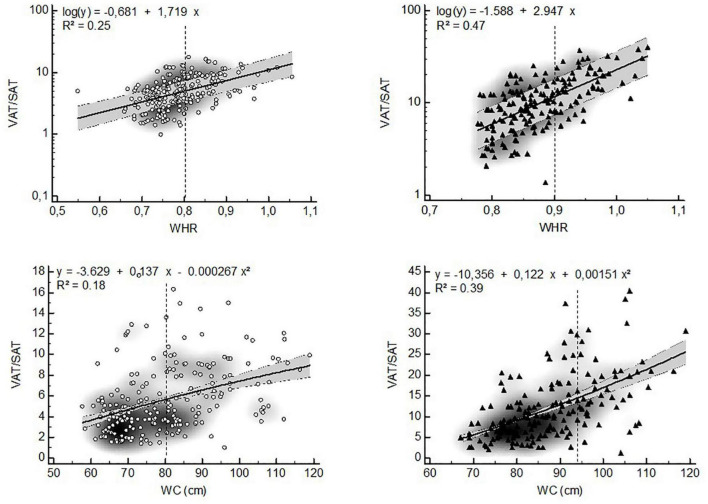
Non-linear relationships between the VAT/SAT ratio and anthropometric estimates of visceral adiposity (▲ = women, ▲ = men, VAT = visceral adipose tissue, SAT = subcutaneous adipose tissue, WHR = waist-to-hip ratio, WC = waist circumference, dashed lines represent WHO (25) cut points for increased cardiometabolic risk in whites, gray zone represents 95% confidence interval).

**FIGURE 4:**
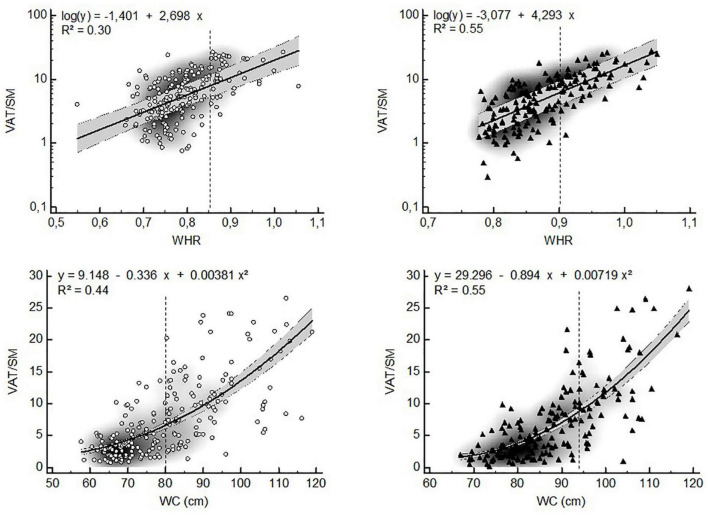
Non-linear relationships between the VAT/SM ratio and anthropometric estimates of visceral adiposity (▲ = women, ▲ = men, VAT = visceral adipose tissue, SM = skeletal muscle, WHR = waist-to-hip ratio, WC = waist circumference, dashed lines represent WHO (25) cut points for increased cardiometabolic risk in whites, gray zone represents 95% confidence interval).

The VAT distribution ratios were non-linearly related to anthropometry-based markers of central obesity in both sexes (Figures 3, 4). The ratio of VAT/SAT showed an exponential relationship with WHR (women: *R*^2^ = 0.25; men: *R*^2^ = 0.47) and a quadratic relationship with WC (women: *R*^2^ = 0.18; *R*^2^ = 0.39) (Figure 3). In both sexes, VAT/SM was exponentially related to WHR (women: *R*^2^ = 0.30; men: *R*^2^ = 0.55). The ratio of VAT/SM showed a quadratic relationship to WC (women: *R*^2^ = 0.44; men: *R*^2^ = 0.55) (Figure 4).

## Discussion

The main finding of this study is that visceral adipose tissue distribution is non-linearly related to age and to anthropometry-based estimates of visceral adiposity. Non-linear relationships between single BC compartments and age have been reported earlier and can be visualized by for example the Gaussian-like distribution for change in adiposity during life (32). Further, muscle and age show a curvilinear relationship due to the increasing rate in muscle loss with advanced aging (40). When used as indices, however, BC ratios (e.g., VAT/SAT) are often assumed to be linearly related to age or other BC components.

**FIGURE 5:**
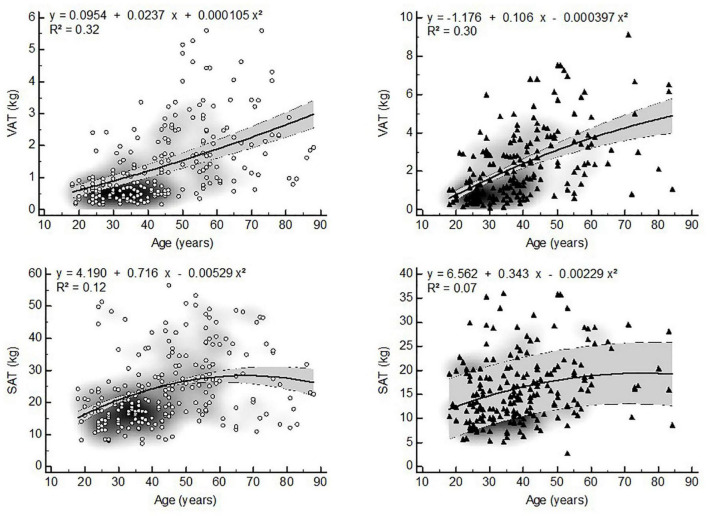
Non-linear relationships between VAT, SAT and age (▲ = women, ▲ = men, VAT = visceral adipose tissue, SAT = subcutaneous adipose tissue, gray zone represents 95% confidence interval).

Although it has previously been reported that the ratio of VAT/SAT increases in a quasi-linear manner with age (34), we found quadratic relationships in both sexes. This can be explained by the fact that the slopes of the increase in VAT and SAT are significantly different between these compartments (Figure 5). Moreover, in old age the VAT compartment continues to increase slowly, while the SAT compartment starts to decrease slowly. Thus, the rate at which both BC compartments change differs considerably in time. As a result, the change in VAT/SAT distribution by age is more pronounced in middle adulthood than in old age.

To the knowledge of the authors, no studies were found describing the relation of the VAT/SM ratio to age. Similar to VAT/SAT a quadratic relationship between VAT/SM and age was found in both sexes. Muscle mass starts to decrease at an age of about 50 years old and accelerates to decrease with advancing age. As a result, the change in VAT/SM shows a similar pattern to the change in VAT/SAT during life, although the former seems to change in a more pronounced way in old age. These results may therefore support the pathophysiological model whereby visceral adiposity accumulation in middle adulthood induces skeletal muscle insufficiency in old age, which in turn may possibly lead to the development of metabolic syndrome or sarcopenic visceral obesity (6, 41).

The changes in WHR and WC over time in the present study are in agreement with previous findings (32, 25). Waist and hip circumferences gradually increase with age until about 70 years old in both sexes. In older age (> 70 years) WHR and WC may further increase at slow rates of about 1-2% per decade in stable-weight subjects or decrease slowly in subjects losing weight (42–44). Since the changes in visceral adipose tissue distribution are more accentuated than the changes in circumferences with age, our results suggest that the latter may underestimate changes in visceral adipose tissue distribution especially in older persons.

Our results show that VAT distribution ratios are exponentially related to WHR and WC. This implies that BC proportions may change more rapidly than proxies of visceral adiposity, especially above the WHO cut points for increased risk. As a result, when used as surrogates for BC, WHR and WC should be corrected according to the risk category. For example, in men a WC of 120 cm corresponds to a VAT/SM ratio that is four times higher than the one at a waist of 90 cm (Figure 3). Of note, the VAT/SM ratio is similar across sexes below the cut points for increased metabolic risk based on WC. However, above these cut points the same increase in WC corresponds to a larger increase in VAT distribution in men compared to women, also highlighting the sex-specific differences in BC.

A recent pilot study showed that both VAT/SAT ratio and WHR correlate with pro-inflammatory cytokines in obese women, suggesting their potential role in the development of cardiometabolic dysfunction (45). Our study showed that VAT/SAT correlated best with WHR in both sexes. This is in contradiction with the findings of Tresignie et al. (46) and Bazzocchi et al. (47) who found no association between VAT/SAT ratio and WHR. Although our results are partially in agreement with those of Seidell et al. (48) who found a positive relation between VAT/SAT and WHR in women, the differences with other studies may be explained by the differences in the assessment of SAT. While it is common to calculate SAT from a medical imaging scan slice using CT or MRI, whole body SAT was used in the present study. Nevertheless, our findings may suggest that hip circumference better reflects whole body adiposity rather than regional (trunk) adiposity in healthy adults.

The VAT/SM ratio correlated best with WC in both sexes. Although most studies agree that redistribution of visceral adiposity occurs after menopause due to the absence of estrogens (49), Franklin et al. (50) found that menopause did not affect the relative abdominal adipose tissue distribution (VAT vs. SAT and VAT vs. muscle) or WC in their longitudinal study. As such, it is possible that the changes in body composition occurring directly after menopause may be captured by WC.

As pointed out, non-linear relationships between BC components have been described previously (32). Despite their different development rates during life, cross-sectional and longitudinal research in adult BC often assumes linear relationships between different levels of organization in BC. Since the interrelationship between these levels of organization might be more complex than usually assumed due to biological variation, their translation toward clinical practice remains challenging. This may be highlighted by the fact that the same amount of change in WC, may represent a variable change in BC components depending on age, sex and absolute quantities. In the field of BC there is a need to establish minimally important clinical differences (MICD), as it is unknown by which amount body components/proportions should change in order to make a difference to the patient. This approach may also help in the operational definition of BC related pathologies, such as cardiometabolic risk or sarcopenic visceral obesity. Future BC research should therefore also focus on determining the smallest change (at different levels of organization) in the treatment of individual patients with similar pathologies as MICD is depending on the BC components that are measured and their respective quantities. By doing so the cross-talk between different BC components in metabolic syndrome and/or sarcopenic visceral obesity may be further elucidated.

### Limitations

There are a number of limitations to consider when interpreting our study results. First, WC was taken midway between the iliac crest and lower rib border. Since the absolute value of WC may differ up to 20% according to its measurement site, our results may only be valid for identical measurement protocols (51).

Secondly, although we described the relation between BC and age, our data are cross-sectional. As longitudinal studies are more accurate in assessing age-related changes in BC than cross-sectional studies do, our results may underestimate the effects of aging on VAT distribution. Therefore longterm follow-up studies assessing VAT distribution changes over time are necessary to confirm our findings.

Finally, analysis of covariance (controlling for age, weight and height) showed that the VAT/SM ratio of Asian women is lower compared to that of white and black women. No other differences in body fat distribution or anthropometric estimates were apparent. Since the group of Asian women represents 13% of the total sample, we assumed that the impact of ethnic differences in body fat distribution may be rather small, albeit it cannot be excluded that the ethnic heterogeneity of our sample might have lowered the predictive accuracy of our regression models.

## Conclusion

Visceral adipose tissue distribution is non-linearly related to age, WHR and WC. These relationships are curvilinear in nature and are influenced by age, sex and quantity. Since WHR and WC may represent different BC compartments, our results also emphasize the challenge when interpreting changes in BC based upon linear extrapolations.

## Data Availability Statement

The raw data supporting the conclusions of this article will be made available by the authors, without undue reservation.

## Ethics Statement

The studies involving human participants were reviewed and approved by institutional review boards of St. Luke’s/Roosevelt Hospital (NY) and Queen’s University (ON). The patients/participants provided their written informed consent to participate in this study.

## Author Contributions

AS designed research (project conception, development of overall research plan, and study oversight), analyzed data or performed statistical analysis, wrote the manuscript (major contribution), and had primary responsibility for final content. JV, EC, and IB had primary responsibility for final content. SH designed research (project conception, development of overall research plan, and study oversight), conducted research (hands-on conduct of the experiments and data collection), provided essential reagents or provided essential materials (contributed by providing constructs, databases, etc., necessary for research), and had primary responsibility for final content. All authors contributed to the article and approved the submitted version.

## Conflict of Interest

The authors declare that the research was conducted in the absence of any commercial or financial relationships that could be construed as a potential conflict of interest.

## Publisher’s Note

All claims expressed in this article are solely those of the authors and do not necessarily represent those of their affiliated organizations, or those of the publisher, the editors and the reviewers. Any product that may be evaluated in this article, or claim that may be made by its manufacturer, is not guaranteed or endorsed by the publisher.

## Footnotes

1 https://www.medcalc.org; 2021.
